# A Comprehensive Phylogenetic Analysis of the Scleractinia (Cnidaria, Anthozoa) Based on Mitochondrial CO1 Sequence Data

**DOI:** 10.1371/journal.pone.0011490

**Published:** 2010-07-08

**Authors:** Marcelo V. Kitahara, Stephen D. Cairns, Jarosław Stolarski, David Blair, David J. Miller

**Affiliations:** 1 ARC Centre of Excellence for Coral Reefs Studies and Coral Genomics Group, James Cook University, Townsville, Queensland, Australia; 2 Department of Invertebrate Zoology, National Museum of Natural History, Smithsonian Institution, Washington, D. C., United States of America; 3 Institute of Paleobiology, Polish Academy of Science, Twarda, Warsaw, Poland; 4 School of Marine and Tropical Biology, James Cook University, Townsville, Queensland, Australia; American Museum of Natural History, United States of America

## Abstract

**Background:**

Classical morphological taxonomy places the approximately 1400 recognized species of Scleractinia (hard corals) into 27 families, but many aspects of coral evolution remain unclear despite the application of molecular phylogenetic methods. In part, this may be a consequence of such studies focusing on the reef-building (shallow water and zooxanthellate) Scleractinia, and largely ignoring the large number of deep-sea species. To better understand broad patterns of coral evolution, we generated molecular data for a broad and representative range of deep sea scleractinians collected off New Caledonia and Australia during the last decade, and conducted the most comprehensive molecular phylogenetic analysis to date of the order Scleractinia.

**Methodology:**

Partial (595 bp) sequences of the mitochondrial cytochrome oxidase subunit 1 (CO1) gene were determined for 65 deep-sea (azooxanthellate) scleractinians and 11 shallow-water species. These new data were aligned with 158 published sequences, generating a 234 taxon dataset representing 25 of the 27 currently recognized scleractinian families.

**Principal Findings/Conclusions:**

There was a striking discrepancy between the taxonomic validity of coral families consisting predominantly of deep-sea or shallow-water species. Most families composed predominantly of deep-sea azooxanthellate species were monophyletic in both maximum likelihood and Bayesian analyses but, by contrast (and consistent with previous studies), most families composed predominantly of shallow-water zooxanthellate taxa were polyphyletic, although Acroporidae, Poritidae, Pocilloporidae, and Fungiidae were exceptions to this general pattern. One factor contributing to this inconsistency may be the greater environmental stability of deep-sea environments, effectively removing taxonomic “noise” contributed by phenotypic plasticity. Our phylogenetic analyses imply that the most basal extant scleractinians are azooxanthellate solitary corals from deep-water, their divergence predating that of the robust and complex corals. Deep-sea corals are likely to be critical to understanding anthozoan evolution and the origins of the Scleractinia.

## Introduction

Although principally known as the architects of coral reefs, the order Scleractinia, or stony corals, comprises two distinct ecological groups: the zooxanthellate species that live in symbiosis with a photosynthetic dinoflagellate occur in shallow tropical waters; and the azooxanthellate species, which are primarily associated with deeper and colder waters. Of the approximately 1490 valid extant scleractinian species [1], more than 47% are azooxanthellate [1], [2] and occur from polar [3], [4] to equatorial regions, and from shallow to bathyal depths [5].

Scleractinians are first known in the fossil record as shallow-water forms from the Middle Triassic (ca. 245 Ma), but by this time were already highly diverged at the subordinal level [6]. However, the small number of reliable skeletal characteristics and the uncertain impact of environmental variables on these morphological characters [7] have severely hampered attempts to infer relationships among families and suborders [8], [9], [10], [11].

Traditionally the inference of evolutionary relationships among corals has relied heavily on comparing extant and fossil material in terms of micro- and macromorphological skeletal characteristics, but this has resulted in several very different schemes [6], [12], [13], [14]. Attempts to establish phylogenetic relationships within coral families based on skeletal characteristics have proved to be challenging, and as a consequence have been applied to date to only six of the 27 extant families – Fungiidae [15], [16], Mussidae [17], [18], Siderastreidae [17], Turbinoliidae [19], Acroporidae [20] and Dendrophylliidae [21].

During the last two decades, there have been various attempts to infer coral phylogeny based on molecular sequence data independent of skeletal morphology. To date, a wide range of markers have been used, both mitochondrial [8], [10], [11], [22], [23], [24], [25], [26], [27], [28] and nuclear [8], [11], [24], [25], [26], [28], [29], [30], [31], [32]. However, these studies imply quite different evolutionary scenarios for scleractinians, particularly in terms of relationships between suborders and families [8], [22]. Furthermore, solitary azooxanthellate species have rarely been included in these analyses, despite accounting for approximately a third of the extant scleractinian species [1], [33].

In an attempt to address these sampling biases and resolve some of the taxonomic uncertainties, we have undertaken the most comprehensive molecular phylogenetic study of the Scleractinia to date. Molecular sequence data were obtained for a ∼590 bp fragment of the mitochondrial cytochrome oxidase subunit 1 gene for 65 deep-sea azooxanthellate scleractinian species collected off New Caledonia and Australia, representing 25 genera and 9 families. With the inclusion of 11 novel sequences from shallow-water corals kindly provided by Dr. Hironobu Fukami (Kyoto University) and 156 additional sequences from GenBank, the dataset covered all of the scleractinian suborders, comprising a total of 234 species from 104 genera representing 25 of the 27 extant families. Unfortunately, we were unable to include representatives of the families Guyniidae and Schizocyathidae in our analyses; these are small (comprising a total of only four monotypic genera) families of deep-sea corals for which material appropriate for molecular analyses rarely becomes available due to their minute size (sometimes less than 2 mm in calicular diameter). Database sequences for corallimorpharians (11 species), actiniarians (2 species), zoanthids (3 species), an antipatharian, and octocorals (4 species) were also included in the analyses as outgroups. The results imply that most families composed predominantly of deep-sea azooxanthellate taxa (Gardineriidae, Micrabaciidae, Flabellidae, Dendrophylliidae, Fungiacyathidae, and Turbinoliidae) are monophyletic, but the caryophylliids and anthemiphylliids, as well as most of the shallow-water zooxanthellate families, require revision.

## Results

The advantage of using CO1 sequence data for coral phylogeny is that, unlike the 16S rDNA, 12S rDNA, and 28S rDNA genes, the sequences are unambiguously alignable because they contain no indels. In addition to 234 scleractinian species, our analysis also included representatives of each of the anthozoan subclasses with the exception of Ceriantharia. The list of all sequences used in the present study is available as File S1. The saturation test showed that there was no significant saturation (*P*<0.0001; *Iss*<*Iss.c*) in the CO1 alignment. Sh-like returned likelihood value of −12912.35, and the Bayesian convergence diagnostic returned a potential scale reduction factor between 1.000 and 1.005, and −13457.44 as the arithmetic mean of the likelihood values between the four runs. Bayesian analyses were also conducted based on the same alignment, but excluding either the third codon position or excluding all transversions, and after translation. All of these kinds of analyses resulted in phylogenies with lower resolution than those based on the full nucleotide sequences, in each case generating large polytomies for the robust shallow water corals. The Bayesian bipartitions of taxon were analyzed for the original run, but none of the generations retrieved a monophyletic Faviidae, Merulinidae, Pectiniidae, or Mussidae family.

Forcing monophyly upon the robust shallow-water coral families resulted in significantly worse likelihood scores than in the absence of constraint (data not shown), implying that, large taxonomical revisions should be carried out.

**Figure 1:**
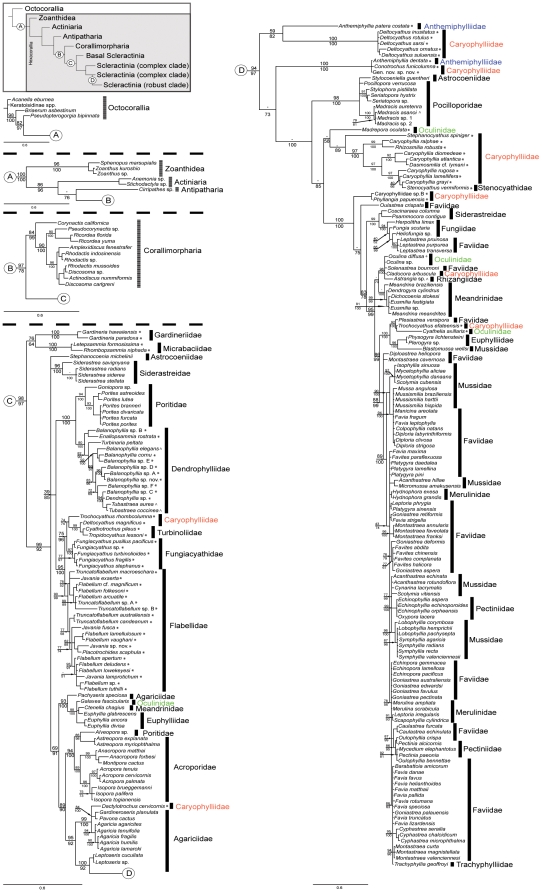
Phylogenetic analyses based on Bayesian inference and Maximum likelihood of the partial mitochondrial CO1 gene from 234 scleractinian species, 11 corallimorpharians, 2 actiniarians, 3 zoanthids, 1 antipatharian, and 4 octocorallians. Topology was reconstructed under the GTR+I+G model of nucleotide evolution in MrBayes. Numbers on branches show Sh-like support (top) calculated using PhyML, and posterior probability (bottom) calculated using MrBayes. Hyphen (−) indicates no support from the respective method. (A) Zoanthids, actiniarians, and antipatharian clade. (B) Corallimorpharian clade. (C) “Basal” and “complex” scleractinian clades. (D) “Robust” scleractinian clade. Colored names indicate families with azooxanthellate representatives that morphological revisions need to be carried out. Asterisks indicate azooxanthellate deep-water scleractinians, carets indicate azooxanthellate shallow-water scleractinians, and plus signs indicate facultative scleractinians.

The results of phylogenetic analyses are summarized in figure 1; the (four) octocoral sequences were used to root the phylogenetic tree because of the sister group relationship between hexacorallians and octocorallians [29], [31], [34], [35], [36]. Maximum likelihood and Bayesian analyses strongly supported monophyly of both Scleractinia and Corallimorpharia (Fig. 1–B) [11], [37], and therefore contradict the “naked corals” hypothesis [27], which suggested that corallimorphs are descended from scleractinians via skeleton loss. In contrast to previous studies [24], Antipatharia were not basal within the Hexacorallia in our analysis. Note, however, the relatively weak support for the position of Antipatharia in our tree.

Within the Scleractinia, the most deeply diverging clade was composed of members of Gardineriidae and Micrabaciidae, two exclusively solitary and azooxanthellate coral families. The overall shape of the remainder of the scleractinian tree is that the “robust” coral clade branches from within the “complex” corals. However, some morphologically defined families are split between these two major groups as documented in several previous papers [8], [10], [11], [32] - the families Astrocoeniidae, Siderastreidae, Oculinidae, Meandrinidae, Euphylliidae, and Caryophylliidae have representatives within both the “complex” and “robust” corals. In addition to members of these families, the “robust” clade comprises Anthemiphyllidae*, Pocilloporidae, Stenocyathidae, Faviidae*, Fungiidae, Mussidae*, Trachyphylliidae, Merulinidae*, Rhizangiidae, and Pectiniidae*. The “complex” coral clade consists of representatives of families Agariciidae*, Acroporidae, Poritidae*, Dendrophylliidae, Flabellidae, Turbinoliidae and Fungiacyathidae in addition to the six families that are split across the “robust/complex” divide. Some families and suborders appear to urgently require revision; those indicated above by asterisks are paraphyletic within the complex or robust clades, whereas oculinids and caryophylliids are paraphyletic within the robust corals as well as in the complex clade.

Nucleotide composition did not differ significantly between sequences in the “complex” and “robust” clades, with %(A+T) mean composition of 61.7% and 67.9%, respectively, and the basal scleractinian clade likewise did not differ significantly from the “complex” or “robust” clade (Table 1). The average difference between sequences within each scleractinian clade was no more than 8%, and within the corallimorpharian clade was 4%, but between “robust” and “complex”, “robust” and “basal”, and “robust” and corallimorpharian clades the corresponding values were 19.1%, 20.1%, and 19.6%, respectively. Members of the “complex” clade displayed an average of 12.3% differences with those of the “basal” clade, and 13.2% differences with corallimorpharian sequences. In total, 27.4% of bases were invariant across the Scleractinia, the transition: transversion ratio was 2.21, and the average difference compared to corallimorpharian sequences was 17.4% (Table 1).

**Table 1:** Nucleotide composition, proportion of invariant sites (Pinv), transition vs transversion rate (Ts/Tv), average distance between sequences (DS), and average distance between clades calculated based on GTR+I+G evolution model.

Clades	Nucleotide composition (%)				Pinv (%)	Ts/Tv	DS (%)	Average distance between clades (%)			
	A	T	C	G				R	C	B	S
R	22.8	39.1	15.0	22.9	32.5	2.084	8	-	-	-	-
C	22.7	39.0	16.8	21.3	33.6	2.565	8	19.1	-	-	-
B	22.0	35.9	18.0	23.9	69.8	2.954	8	20.1	12.3	-	-
S	22.7	38.7	15.7	22.7	27.4	2.210	13	-	-	-	-
Co	23.4	35.7	17.5	23.3	35.1	2.666	4	19.6	13.2	13.2	17.4
A	24.1	37.6	16.7	21.5	28.8	2.354	14	-	-	-	-

R =  “Robust” scleractinian clade.

C =  “Complex” scleractinian clade.

B =  “Basal” scleractinian clade.

S =  Scleractinia clade (robust + complex + basal).

Co =  Corallimorpharia clade.

A =  All alignment (including Octocorallia, Antipatharia, Zoanthidea, Actiniaria, Corallimorpharia, Scleractinia).

For some genera, the molecular phylogeny is inconsistent with family placements based on classical taxonomy, implying that the positions of these should be re-evaluated. This category includes the azooxanthellate genera *Conotrochus*, *Madrepora*, *Stenocyathus*, *Phyllangia*, *Cladocora, Trochocyathus,* and *Dactylotrochus,* as well as the zooxanthellate genera *Pachyseris*, *Galaxea*, *Ctenella*, *Alveopora*, and most of the “robust” coral representatives.

## Discussion

Both maximum likelihood and Bayesian analyses support the distinction of two major clades (“complex” and “robust” corals) within the Scleractinia (Fig. 1–C and D), as was previously implied by molecular analyses based on mitochondrial 16S rDNA [8], [10], [22], [23], 12S rDNA [25], and CO1 + Cyt B data [11], and on (nuclear) 28S rDNA sequences [11], [29], [30], [32]. However, rather than the deep split between these two groups implied by analyses of ribosomal sequences, our phylogeny places the “robust” coral clade within the “complex” radiation, following the precedent of Fukami *et al*. [11]. This topology has high Sh-like (ML) and posterior probability (BI) support, and was not significantly affected by weighting the analyses for codon position.

Our analyses imply that the families Gardineriidae and Micrabaciidae, which are exclusively azooxanthellate and contain only solitary species, represent the most basal lineage of modern scleractinians, supporting the concept that deep-sea corals hold important clues regarding the evolutionary history of the order. The evolutionary implications of the basal position of gardineriids and micrabaciids are more fully explored elsewhere (Stolarski *et al*., in preparation), however, the basal position of these families suggests that the ancestral scleractinian may also have been solitary and azooxanthellate. According to Owens [38] and Squires [39], ancestral micrabaciids probably inhabited shallow-water environments but may have been essentially preadapted for deep-sea life by having auto-mobile coralla [38], and thus been able to gradually invade deeper waters, resulting in an increase of skeleton porosity [39]. Similarly, fossils thought to represent the oldest known gardineriid (*Rodinosmilia elegantula*) were described from Morocco [40], suggesting that this family may also have first appeared in shallow-water environments. Under this scenario, the early Mesozoic appearance of diverse, highly integrated colonial forms may reflect the advent of symbioses with the dinoflagellate *Symbiodinium*, as has been suggested based on stable isotope data [41]. Since all early Mesozoic records of Scleractinia represent rather shallow-water ecological settings, it is not yet possible to infer whether the Scleractinia were initially abyssal and then colonized shallow waters (as hypothesized by Lindner et al. [42] for the stylasterid corals), or vice-versa.

Recent molecular analyses are inconsistent with widely used sub-ordinal classification schemes of Vaughan and Wells [43] and Wells [6], which were based on morphology. Although morphological support for the “robust” and “complex” dichotomy is still lacking, it is consistently supported by molecular analyses and the three clades recovered here (“basal”, “complex”, and “robust”) could represent a new sub-ordinal scheme for the classification and evolutionary history of the order.

One general implication of the phylogenetic analyses reported here is that the majority of the azooxanthellate coral families (six of the eight) are monophyletic, whereas only a minority of families (four of seventeen) that are predominantly or exclusively zooxanthellate are supported strongly by the molecular data. Thus many of the morphologically defined families of shallow-water corals do not represent “natural” families. This conclusion is broadly consistent with Fukami *et al*. [11], although this work was based on more limited sampling of azooxanthellate corals. Below we discuss the status of some individual families based on the overall CO1 phylogeny.

### Flabellidae

The flabellids are a large family of exclusively azooxanthellate corals that formed a single well-supported clade in our analyses, which were based on 27 species representing the full morphological spectrum of the family (only missing genera with root-like structures e.g. *Rhizotrochus*). Interestingly, the CO1 analyses suggest that there may be a major dichotomy within the family, with representatives of many genera examined occurring in both of the resulting clades. The general pattern is that *Truncatoflabellum* species occupy basal positions in both clades, with different *Javania* species branching next. These results suggest that the relationship of flabellids with substrata and their mode of reproduction diversified during their evolution. To date we can infer that the most basal form of substrata relationship and reproduction within the extant flabellid genera is fixed (fragile pedicel), with transverse division as the main reproduction mode respectively (as observed in *Truncatoflabellum*). Subsequently, multiple, concentric layers of sclerenchyme reinforcing the pedicel and the attachment of the corallum to substrate (as observed in *Javania* and being a trait also shared with the anthocaulus of *Placotrochides*) appears in our analysis. Later, the substrate relationship became less evident, or present only in very early developmental stages, with adult specimens becoming free-living forms, such as observed within *Flabellum*. The evolutionary position of different root-like attachment structures present in other flabellid genera (e.g. *Monomyces*, *Polymyces*, *Rhizotrochus*) needs to be further investigated.

The analyses imply a close relationship between the Flabellidae and two other exclusively azooxanthellate coral families – Turbinoliidae and Fungiacyathidae. Both monophyly of Flabellidae and the relationship between this family and Turbinoliidae and Fungiacyathidae are consistent with previous work of Le Goff-Vitry *et al*. [10].

### Fungiacyathidae and Turbinoliidae

The five fungiacyathid representatives sequenced formed a well-supported group notwithstanding the method used, corroborating their family status [13]. In addition to the link with Flabellidae and Fungiacyathidae outlined above, our analyses imply a close relationship of Turbinoliidae with two caryophylliids – *Trochocyathus rhombcolumna* and *Deltocyathus magnificus*. Additional material is necessary to better understand the relationships within the turbinoliids, as only two species representing two genera are present in our phylogeny. To collect fresh turbinoliids is particularly challenging because they are among the smallest known scleractinians. The turbinoliids *Cyathotrochus pileus* and *Tropidocyathus lessoni* grouped with *D. magnificus* sharing a common ancestor with *T. rhombcolumna*. Morphological support for this grouping is, however, lacking. Similarity of the CO1 sequences between *Deltocyathus magnificus* (four specimens from different collecting stations sequenced) and turbinoliids is difficult to explain although they do share some morphological characters (e.g. lamellar paliform lobes before all but last septal cycle forming a chevron arrangement - not fusing in *Tropidocyathus* but fusing in *Deltocyathus* and *Cyathotrochus*, corallum invested with soft tissue, well developed costae, and a papillose columella). All other *Deltocyathus* representatives sequenced in the present study grouped in a basal position in the “robust” clade, and could represent a distinctive family once the other caryophylliid species have been separated into five distinct clades.

### Dendrophylliidae

With nearly 170 species [21], Dendrophylliidae is the third most speciose family of extant scleractinians and in our analyses was the only well-supported family with substantial representation of both shallow and deep-water species. Within the family, a clade comprising the deep-sea colonial species *Enallopsammia rostrata* and a solitary deep-sea *Balanophyllia* sp. diverged most deeply, followed by the shallow-water zooxanthellate colonial genus *Turbinaria.* Representatives of the azooxanthellate genera *Dendrophyllia* (identification needs to be re-evaluated), *Tubastraea*, and *Balanophyllia*, the first two of which are colonial and the last solitary, appear as most recently diverged. The topology is consistent with an azooxanthellate dendrophylliid ancestor, and the possibility of multiple gains or losses of the colonial state within the family. Dendrophylliids are a particularly interesting group and could be highly informative with respect to the evolution of coloniality and the symbiotic state.

### Poritidae and Acroporidae

The families Poritidae and Acroporidae are the most speciose and diverse of shallow-water scleractinians, and are exclusively colonial and zooxanthellate. Our analyses support that the poritid genus *Alveopora* (the only poritid genus with septa not formed by 3 to 8 nearly vertical trabeculae) should be transferred to the Acroporidae (Fig. 1), as the single *Alveopora* sequence grouped with those from *Astreopora explanata* and *Astreopora myriophthalma* within the well-supported acroporid clade [10], [11]. If *Alveopora* is transferred to acroporids, Poritidae becomes monophyletic, as the remaining poritid genera (*Goniopora* and *Porites*) form a well-supported clade.

The molecular phylogeny (Fig. 1) implies a sister group relationship between dendrophylliids and poritids, the latter of which is one of the few families of zooxanthellate corals to have strong support in our analyses. The common ancestry of Poritidae and Dendrophylliidae implied by our analyses is consistent with previous molecular analyses based on 28S rDNA [30], 16S rDNA [8], [23], and the nuclear rDNA, CO1 and Cyt B [11]. The earliest record of poritids is from the Mid-Cretaceous [6], and for dendrophylliids the Early Cretaceous [44]. Veron [14] suggested that the (Late Cretaceous) Actinacididae might be ancestral to the poritids. However, based on macro and microstructures of the skeleton, Cairns [21] advocated that the actinacidids were probably not the dendrophylliid ancestor.

In common with previous molecular analyses [8], [10], [11], [25], the family Acroporidae was monophyletic in our CO1 analyses. Within the Acroporidae, *Anacropora* appears to be more related to *Montipora*, and *Acropora* to *Isopora*, which was recently elevated to genus level [45].

### Agariciidae

The family Agariciidae occupies a special position in our analyses, as the entire “robust” coral clade branches from within a clade that captures the agariciids (excluding *Pachyseris speciosa*) together with the caryophylliid genus *Dactylotrochus*. The Caryophylliidae is not a valid family, its members are scattered throughout the phylogenetic tree (see below). There is morphological support for transferring *Dactylotrochus* to the Agariciidae–for example, the shared presence of highly developed septal menianae (Kitahara *et al*., in preparation; also see [46]). Agariciids are shallow water, colonial corals. Whilst this transfer would make *Dactylotrochus* the only exclusively solitary (and azooxanthellate) extant member of the family, there are precedents from the Cretaceous; the fossil agariciids *Vaughanoseris* and *Trochoseris* were solitary. The latter is recorded from the Late Cretaceous and Paleocene of Saudi Arabia and Pakistan respectively [47], and could represent a genus related to *Dactylotrochus*.

In our analyses, the agariciid clade formed by representatives of *Gardineroseris*, *Pavona*, and *Agaricia* was strongly supported whereas, pending morphological confirmation, *Pachyseris speciosa* may be transferred to euphyllids. A number of other recent analyses [8], [10], [11], [32], [48] also implied monophyly of the Agariciidae.

### Meandrinidae, Astrocoeniidae and Anthemiphylliidae

Unlike the situation with the “complex” corals, where morphology and molecular data are broadly consistent in support of many families, in the case of “robust” corals, the opposite is true. With the sole exception of the Pocilloporidae, every robust family was para- or polyphyletic in our analyses.

In the case of meandrinids, the Atlantic genera (*Meandrina*, *Dichocoenia*, *Dendrogyra*, and *Eusmilia*) formed a strongly supported clade, but the only non-Atlantic meandrinid that we were able to include in the present analysis (*Ctenella chagius*) grouped with the euphylliids (see below), challenging the monophyly of this small family. Clarifying the status of Meandrinidae will require data for additional Indo-Pacific genera; *Gyrosmilia* and *Montigyra*, both transferred to the family [49] are of particular interest.

Only two members of the family Astrocoeniidae were included in our analyses, the Atlantic species *Stephanocoenia michelinii* and the Indo-Pacific species *Stylocoeniella guentheri;* the former fell into the “complex” clade and the latter in the “robust” clade. The fossil record implies an early origin for the family; astrocoeniid-like corals with styliform and vertically continuous columella from the Middle Triassic [6] are amongst the oldest scleractinian fossils yet found. Our analysis supports the idea that *Stylocoeniella* is related to pocilloporids [11]: *S. guentheri* forms a strongly supported group with *Pocillopora*, *Stylophora*, *Seriatopora*, and *Madracis*, and this clade diverges near the base of the radiation of “robust” corals (Fig. 1). To date, no sequence data are available for *Palauastrea*, which was suggested to belong to astrocoeniids [49], and to pocilloporids by Yabe & Sugiyama [50].

Although Anthemiphylliidae affinities are as yet unclear, our analyses support an early divergence of *Anthemiphyllia patera costata* in the “robust” coral clade. Described to accommodate the genus *Anthemiphyllia*, which according to Vaughan [51] “had puzzled every student since its description”, this family is composed of seven species and two subspecies, all with free and solitary growth form, and lobate to laciniate septal edges. Of the eight *Anthemiphyllia* morphs, only *A. patera patera* is exclusively Atlantic, the seven other morphs occurring mainly in Pacific waters (with exception of *A. dentata,* which is recorded also in Indian Ocean waters [52]). If the basal position of *A. patera costata* (and presumably *A. patera patera*) holds with other genetic markers, it may represent that the common anthemiphylliid ancestor was morphologically very close to the extant *A. patera* morphs, and probably inhabited the Tethys Sea 65 Mya. However, it is difficult to understand why *Anthemiphyllia dentata* did not group with *A. p. costata*, considering that all anthemiphylliids share skeletal micro-structural characters (Stolarski, unpublished data).

### Caryophylliidae

The family Caryophylliidae is the least cohesive of extant coral families, as it is represented in distinct clades in both the complex and robust parts of the tree. The affinity of *Dactylotrochus cervicornis* with agariciids, and that of *Deltocyathus magnificus* and *Trochocyathus rhombcolumna* with turbinoliids and other complex corals have been discussed above. In addition, most members of the genus *Deltocyathus* form a distinct clade of uncertain affinity.

One substantial grouping within Caryophylliidae comprises all of the *Caryophyllia* species, *Stenocyathus vermiformis*, *Dasmosmilia* cf. *lymani*, and *Rhizosmilia robusta*; support for association of *Stephanocyathus spiniger* with this clade is weak. Interestingly, the genus *Stenocyathus*, which is one of the two genera assigned to the recently proposed family Stenocyathidae, groups with strong statistical support with *Caryophyllia grayi*, *C. lamellifera*, and *C. rugosa* (also see [53]). This result corroborates the hypothesis that thecal pores originated independently in different scleractinian lineages [54], once *S. vermiformis* is grouping within the “robust” corals, and *Guynia annulata*, another species that has pores groups within the “complex” corals in the 16S rDNA phylogeny (Kitahara et al., unpublished data and [22]). As advocated by Stolarski [54], this hypothesis suggests stability of the basic microstructural architecture of the skeleton, and places the family Stenocyathidae in the superfamily Caryophyllioidea rather than Guynioidea.

The clade formed by *Caryophyllia diomedeae*, *Caryophyllia atlantica*, and *Dasmosmilia* cf. *lymani* also received strong support regardless the method used, and is consistent with the hypothesis that *Dasmosmilia* is a sister genus of *Caryophyllia* [53]. The last representative of the genus *Caryophyllia*, *C. ralphae*, groups with *Rhizosmilia robusta*. *C. ralphae* is one of the most distinctive of *Caryophyllia* species [53], and resembles three other species (*C. capensis*, *C. paucipalata* and *C. eltaninae*) in terms of the placement of paliform lobes [55]. Morphologically, *C. ralphae* is distinguished by its highly exsert septa and very deep fossa, but can be confused with *R. robusta*, both having about the same adult corallum size, septal symmetry and exsertness, colour, and fossa depth. The presence of concentric rings of partitioned chambers in the base cross section of *R. robusta* is one of the few morphological characters that distinguish it from *C. ralphae*. However, the CO1 data demonstrate that the morphological similarity of these two species reflects a close evolutionary relationship. According to Zibrowius & Gili [56], *C. capensis* is not a true *Caryophyllia*, and Cairns [55] suggested that if this species belongs to a different genus, *C. ralphae* should be placed with it. If the genetic relationship between *C. ralphae* and *R. robusta* stands, the presence of the concentric rings of partitioned chamber in the base cross section in the genus *Rhizosmilia* was acquired only recently from a solid-based ancestor.

The placement of *Stephanocyathus spiniger* in the *Stenocyathus*/*Caryophyllia*/*Dasmosmilia*/*Rhizosmilia* clade is unexpected, since the other two representatives of this genus, *S. weberianus* and *S. coronatus* group have quite different affinities on the basis of 16S rDNA sequence analysis (Kitahara *et al*., unpublished data).

Based on the presence of 12–18 short basal tubercles in *Stephanocyathus* (*Odontocyathus*), 6 long costal spines corresponding to each first costae cycle in *S*. (*Acinocyathus*), and no tubercles or spines in *S*. (*Stephanocyathus*), the genus *Stephanocyathus* is divided into the above three subgenera. *S. weberianus* and *S. coronatus* belonging to *S.* (*Odontocyathus*) and *S. spiniger* to *S.* (*Acinocyathus*). If the segregation of *Stephanocyathus* subgenus is detected with one molecular marker, not the case presented above, which is the comparison between 16S rDNA phylogeny for *Odontocyathus* (Kitahara *et al*., unpublished data), and CO1 phylogeny for the *Acinocyathus*, it may indicate that the subgenus should be elevated to genus status (belonging to different families). Their macro-morphological similarity (if the genetically distance between them is confirmed) could be an evolutionary throwback, such as phenotypic characters preserved in DNA reappearing through different lineages from the same ancestor.

Another caryophylliid genus that needs re-evaluation regarding hierarchical status is the exclusively azooxanthellate *Phyllangia*. The basal position of this genus regarding almost all “robust” shallow water corals can represent an azooxanthellate shallow-water ancestor for them (the genus *Phyllangia* is reported exclusively from waters shallower than 100 m [57]).

### Siderastreidae

Another family that has representatives within both major clades is the exclusively shallow water Siderastreidae. The genus *Siderastrea* (represented in our analysis by three Atlantic species: *S. radians*; *S. siderea*; and *S. stellata*, and by the Indo-Pacific *S. savignyana*) forms a well-supported clade within the “complex” corals. Nonetheless, representatives of *Coscinaraea* and *Psammocora* form a clade within the “robust” corals sharing the same common ancestor with the massive faviid genera *Leptastrea* and large solitary fungiids *Heliofungia*, *Fungia* and *Herpolitha*. Combined CO1 and Cyt-B analysis [11] also recovered this clade, but nuclear phylogenies from the same study did not. The position of *Oulastrea crispata* within the “robust” corals and its relationship to the “robust” siderastreids and fungiids did not receive good statistical support from ML and BI.

Using 16S rDNA sequences, Romano & Palumbi [23] and Romano & Cairns [8] also found that *Coscinaraea*, *Psammocora*, *Fungia*, and *Leptastrea* are closely related. Partial 5.8S and ITS2 sequences, and skeletal microstructure analysis clearly suggest that *Psammocora* and *Coscinaraea* are closer to fungiids than to siderastreids, however, both genera are not monophyletic [58]. According to the same study, the genus *Pseudosiderastrea* grouped with the “*Siderastrea*” clade.

The “siderastreids” that clustered in the “robust” coral clade are distinct from *Siderastrea* on the basis of both morphology [17] and molecular data, and probably do not belong to this family, once the type genus of this family was established as *Siderastrea* [43]. Forsman *et al*. [59] also concluded that the Atlantic species of *Siderastrea* form a monophyletic group, and *S. glynni* (the only Eastern Pacific representative of this genus) also appears to be closed related to the Atlantic species.

### Oculinidae

As in previous studies [8], [10], the oculinids were polyphyletic in our analyses, with *Galaxea* falling into the “complex” clade, and *Madrepora*, *Oculina*, and *Cyathelia* occupying distinct positions within the “robust” clade. The strongly-supported grouping of *Galaxea* with the meandrinid *Ctenella chagius* and the euphyllids *Euphyllia glabrescens*, *E*. *ancora*, and *E*. *divisa* seen in our analyses (Fig. 1) support Fukami's [11] suggestion that *Galaxea* and *Ctenella* should be transferred to the Euphylliidae. Le Goff-Vitry *et al*. [10] suggested that the genus *Madrepora* should be elevated to family status; the poorly resolved position of *M. oculata* in our analysis is consistent with this, although the remaining four congeners (*M. arbuscula*, *M. carolina*, *M. minutiseptum*, and *M. porcellana*) need to be examined. In our analysis, strong support was obtained for a clade containing *Oculina* and members of three other families-*Cladocora*, *Solenastrea*, and *Astrangia*–but it is unclear whether this clade has morphological support. The significance of the grouping of *Cyathelia axillaris* with a shallow-water massive faviid and a solitary azooxanthellate caryophylliid is also unclear. Representatives of *Bathelia*, *Petrophyllia*, *Shizoculina*, *Sclerhelia*, and *Simplastrea* have not been sequenced to date, and their position within the oculinids needs to be re-evaluated.

### Other families

One of the most heterogeneous groups formed in our analysis is composed by five different families: Mussidae (*Blastomussa wellsi*); Euphylliidae (*Physogyra lichtensteini* and *Plerogyra*); Caryophylliidae (in part: *Trochocyathus efateensis*); Oculinidae (in part: *C. axillaris*); and Faviidae (*Plesiastrea versipora*). This clade is strongly supported by all phylogenetic methods and agrees with Fukami *et al*. [11] who, excluding *T. efateensis*, recovered the same clade. In fact, the presence of the solitary deep-water azooxanthellate Indo-Pacific species *T. efateensis* within this clade (otherwise all zooxanthellate and colonial) is difficult to explain and requires further investigation. On the basis of 16S rDNA analyses (Kitahara *et al.*, unpublished data) *T. efateensis* groups with two other deep-water caryophylliids (*Trochocyathus cepula* and *Tethocyathus virgatus*). Kitahara *et al*. [53] briefly discussed the relationship between the latter two genera.

Most of the remaining species included in our phylogenetic tree are from exclusively zooxanthellate coral families, and the CO1 data imply that these species diverged relatively recently. Our results are consistent with previously analyses [11], [18], [26], [32], which found that most of these coral families are polyphyletic–most strikingly, phylogenetics often splits Pacific and Atlantic representatives of the same genus or family (see [26]). One of the most highly fragmented families in our analyses is Faviidae, which is split into ten different groups (Fig. 1–D). As reported by Fukami *et al*. [11], the Indo-Pacific faviids appear to be clearly distinct from their Atlantic counterparts, and the latter should probably be transferred to an Atlantic mussid clan/clade, with the following composition: *Isophyllia* spp. *Mycetophyllia* spp., *Mussismilia* spp., *Diploria* spp., *Manicina* spp., *Colpophyllia* spp., *Scolymia cubensis*, *Favia fragum* and *F. leptophyllia* (see also [60]).

According to our results and following Fukami *et al*. [11], the Trachyphylliidae does not merit recognition at the family level and should be incorporated into the Indo-Pacific “faviid-pectinid-merulinid” clan/clade. *Montastraea cavernosa* did not group with its congeners, but rather diverged near the base of the “robust” clade, and few conclusions can be drawn concerning the remaining faviids in our phylogeny.

### Conclusions

Maximum Likelihood and Bayesian analyses of the CO1 data set indicate that most of the exclusively zooxanthellate coral families are not monophyletic, and require morphological revision. By contrast, the majority of families consisting exclusively or predominantly of azooxanthellate corals appears to be monophyletic. An important exception is the azooxanthellate family Caryophylliidae; here, special attention should be given to the genera *Deltocyathus*, *Trochocyathus*, and the heterogeneous group formed by *Stephanocyathus*, *Vaughanella*, *Conotrochus*, *Paraconotrochus*, Gen. nov. A sensu Stolarski (1996), and *Ceratotrochus*.

Whereas the deepest dichotomy identified in previous studies was the complex/robust split, our analyses (the present study and Stolarski *et al*., in preparation) also identified a deeply-diverging clade consisting of members of the exclusively azooxanthellate families Gardineriidae and Micrabaciidae. On the basis of our analyses, these may be the oldest scleractinian families with extant representatives. Although estimates of divergence times among gardineriids/micrabaciids and the complex and robust lineages must be further investigated, the placement of these families as basal to the complex/robust coral lineages implies that scleractinians may have co-existed with rugose corals but, unlike the latter, survived the Permian/Triassic mass-extinction event.

The deep-sea holds important clues to anthozoan evolution, and overall, our phylogenetic reconstruction shows that the most basal extant scleractinians are azooxanthellate corals from deep-water (probably with azooxanthellate shallow-water ancestors), not only in the case of gardineriids/micrabaciids, but also in relation to the “robust” coral clade, and possibly within extant agariciids. Another conclusion can be drawn within the acquisition or loss of solitary/colonial state. Even though most of the groups apparently arouse from solitary life forms, the opposite was also detected (e.g. pocilloporids and *M. oculata* in relation to *Caryophyllia*).

Finally, our data supports that the order Corallimorpharia is the sister group of scleractinians [11], [37] and are therefore inconsistent with the “naked coral” hypothesis, which implies that corallimorphs are corals that have undergone skeleton loss.

## Materials and Methods

Between 1993 and 2007, French and Australian expeditions collected and preserved in ethanol hundreds of specimens of deep-water scleractinians (ranging in depth from 170 to 1434 m) from off New Caledonia and Australian waters (including Pacific and Indian Ocean). Based on morphological characters all these specimens were identified to the lower taxonomic level possible, and genomic DNA was extracted from most of them. The definition used here to delimit the upper depth boundary of deep-water corals is 50 m [1], since very few zooxanthellate corals occur below this depth.

Tissue was collected from a whole mesentery using a forceps when the species was large, or an entire sector (including the skeleton) was taken when the species was small. However, intending to preserve museum vouchers, if just one specimen of a small solitary species was available, the specimen was completely submerged in the lysis buffer to have its genomic DNA extracted. Genomic DNA was extracted using DNeasy Tissue and Blood Kit (QIAGEN) following the manufacturer's instructions. For each species the concentration of genomic DNA extracted was measured using a Nanodrop 1000 (Thermo Scientific), and when necessary, an aliquot of the genomic DNA was diluted or concentrated to achieve the final concentration of 25 ng/ul.

Using the primers developed by Folmer *et al*. [61] (LCO1 490-GGTCAACAAATCATAAAGATATTGG and HCO2 198-TAAACTTCAGGGTGACCAAAAAATCA) a fragment of the mitochondrial cytochrome oxidase subunit 1, ranging between 700 and 710 bp according to the species, was amplified. Reactions were carried out in 50 µl, with 5 µl of 10× PCR Buffer, 5 µl of 2 mM dNTPs, 5 µl of 25 mM MgCl2, 2.5 µl of each primer (10 mM each), 0.4 µl of *Taq* polymerase, and 2 µl of template. PCR conditions used were: a denaturation first step of 95°C for 1 min, followed by 35 cycles of 30 s at 95°C, 30 s at 40°C, and 90 s at 72°C, followed by 10 min at 72°C. If the amplification using this protocol failed, a new reaction using the Advantage-2 kit (Clontech) with the same template, primers and PCR conditions were performed following the manufacturer's instructions. All cycles were performed using Bio-Rad DNA engine (Peltier Thermal Cycler). The PCR products were then purified using Ultra Clean PCR clean up (Mo-Bio) spin columns, and then submitted to Macrogen (Korea) sequencing facility to be sequenced using ABI3730XL (Applied Byosystems). Sequences were verified and manipulated with Sequencher ver. 4.8 (Gene Codes Corporation). A Blast search was performed on GenBank for each sequence and the matching homologous Scleractinian sequences were retained for subsequent alignment. Using this protocol, 158 previously published sequences were added to the alignment (File S1).

All sequences were aligned in ClustalW (EBI) using default settings. The resultant alignment was then checked using JalView ver. 8.0 [62], totaling 595 bp in the final alignment (File S2). The alignment was then submitted to the test of substitution saturation [63] available in DAMBE [64].

Using the final alignment, GTR + Gamma + Proportion Invariant (GTR+G+I) model of DNA evolution was determined by the hierarchical likelihood ratio test implemented in MrModeltest [65] as the best model for the data. The phylogenetic analysis was performed using PhyML for maximum likelihood [66] and MrBayes for Bayesian inference [67], [68].

The most likely topology was calculated based on Shimodaira and Hasegawa (Sh-like) branch support implemented in PhyML. For the Bayesian inference, four runs with 10 million generations each were calculated with topologies saved at each 1000 generations. One fourth of the 10000 topologies were discarded as burnin, and the remaining used to calculate the posterior probability. Additional Bayesian analyses were conducted using BEAST [69] specifically to test the hypothesis that the “robust” shallow water scleractinian families are monophyletic. The BEAST analyses were based on the same alignment as the PhyML and MrBayes phylogenetic analysis, but with and without the constraint of monophyly of the “robust” shallow water scleractinian families.

## Supporting Information

File S1 Species of Scleractinia sequenced for CO1, including station, location of skeletal voucher, and accession number.
(0.36 MB DOC)

File S2 Partial CO1 gene alignment from 255 anthozoans, including 234 scleractinians from 104 genera representing 25 of the 27 extant families.
(9.97 MB TIF)
