# Supplementary material for: A Comprehensive Phylogenetic Analysis of the Scleractinia (Cnidaria, Anthozoa) Based on Mitochondrial CO1 Sequence Data
Source: PLoS One. 2010 Jul 8;5(7):e11490. doi: 10.1371/journal.pone.0011490 (PMC2900217; doi:10.1371/journal.pone.0011490)
Supplement: File S1 — Species of Scleractinia sequenced for CO1, including station, location of skeletal voucher, and accession number. (0.36 MB DOC) [file pone.0011490.s001.doc]

**File S1**. Species of Scleractinia sequenced for CO1, including station, location of skeletal voucher, and accession number.

| Traditional taxonomy | | | Collection locality  OR  Reference | Location of voucher | Accession Number |
| --- | --- | --- | --- | --- | --- |
| Order Scleractinia  Family | *Genus* | *species* |
| Gardineriidae | *Gardineria* | *G. hawaiiensis* | 1 | JCU | GQ868678 |
|  |  | *G. paradoxa* | 1 | JCU | GQ868682 |
| Micrabaciidae | *Letepsammia* | *L. formosissima* | 1 | JCU | GQ868685 |
|  | *Rhombopsammia* | *R. niphada* | 1 | JCU | GQ868683 |
| Dendrophylliidae | *Balanophyllia* | *B. cornu* | SS 102005 stn. 140-16 | JCU | HM018605 |
|  |  | *B.* sp. nov. | SS 102005 stn. 115-48 | JCU | HM018612 |
|  |  | *B.* sp. A | Norfolk 1 stn. DW 1651 | JCU | HM018606 |
|  |  | *B.* *desmophyllioides* | Norfolk 2 stn. DW 2119 | JCU | HM018607 |
|  |  | *B.* sp. C | Norfolk 1 stn. DW 1651 | JCU | HM018608 |
|  |  | *B.* sp. D | Norfolk 2 stn. CP 2141 | JCU | HM018609 |
|  |  | *B.* sp. E | Norfolk 2 stn. DW 2057 | JCU | HM018610 |
|  |  | *B.* sp. F | SS 102005 stn. 096 | JCU | HM018611 |
|  | *Enallopsammia* | *E. rostrata* | Norfolk 2 stn. DW 2075 | JCU | HM018632 |
| Turbinoliidae | *Cyathotrochus* | *C. pileus* | Norfolk 2 stn. DW 2137 | JCU | HM018623 |
|  | *Tropidocyathus* | *T. lessoni* | SS (blank label) | JCU | HM018669 |
| Fungiacyathidae | *Fungiacyathus* | *F. fragilis* | SS 102005 stn. 121-036 | JCU | HM018645 |
|  |  | *F. p. pacificus* | SS 022007 stn. 009-036 | JCU | HM018646 |
|  |  | *F. stephanus* | SS 102005 stn. 149 - 019 | JCU | HM018647 |
|  |  | *F. turbinolioides* | SS 022007 stn. 007-015 | JCU | HM018648 |
| Flabellidae | *Flabellum* | *F. apertum* | SS 022007 stn. 050-024 | JCU | HM018635 |
|  |  | *F. arcuatile* | Norfolk 2 stn. DW 2132 | JCU | HM018636 |
|  |  | *F. deludens* | SS 022007 stn. 008-009 | JCU | HM018638 |
|  |  | *F. folkesoni* | SS 102005 stn. 171-033 | JCU | HM018639 |
|  |  | *F. lamellulosum* | SS 102005 stn. 171-033 | JCU | HM018640 |
|  |  | *F. lowekeyesi* | SS 022007 stn. 041-27 | JCU | HM018641 |
|  |  | *F.* cf. *magnificum* | SS 102005 stn. 172-051 | JCU | HM018637 |
|  |  | *F.* sp. | SS 102005 stn. 017-039 | JCU | HM018642 |
|  |  | *F. tuthilli* | SS 102005 stn. 033-008 | JCU | HM018643 |
|  |  | *F. vaughani* | SS 102005 stn. 130-009 | JCU | HM018644 |
|  | *Javania* | *J. exserta* | Norfolk 2 stn. DW 2162 | JCU | HM018651 |
|  |  | *J. fusca* | Norfolk 2 stn. DW 2064 | JCU | HM018652 |
|  |  | *J. lamprotichum* | SS 022007 stn. 016-015 | JCU | HM018653 |
|  |  | *J.* sp. nov. | Norfolk 2 stn. CH2115 | JCU | HM018654 |
|  | *Truncatoflabellum* | *T. australiensis* | SS 102005 stn. 170-086 | JCU | HM018670 |
|  |  | *T. candeanum* | SS 102005 stn. 115-49 | JCU | HM018671 |
|  |  | *T. macroeschara* | SS 102005 stn. 141-015 | JCU | HM018672 |
|  |  | *T.* sp. A | Norfolk 2 stn. DW 2137 | JCU | HM018673 |
|  |  | *T.* sp. B | SS 102005 stn. 034-056 | JCU | HM018674 |
|  | *Placotrochides* | *P. scaphula* | SS 022007 stn. 007-014 | JCU | HM018661 |
| Anthemiphylliidae | *Anthemiphyllia* | *A. dentata* | SS 102005 stn. 004-067 | JCU | HM018603 |
|  |  | *A. patera costata* | Norfolk 2 stn. DW 2075 | JCU | HM018604 |
| Caryophylliidae | *Caryophyllia* | *C. atlantica* | SS 022007 stn. 050-050 | JCU | HM018613 |
|  |  | *C. diomedeae* | SS 022007 stn. 041-27 | JCU | HM018614 |
|  |  | *C. grayi* | SS 102005 stn. 1??-47 | JCU | HM018615 |
|  |  | *C. lamellifera* | TAN 0308/24 | JCU | HM018616 |
|  |  | *C. ralphae* | Norfolk 2 stn. DW 2135 | JCU | HM018617 |
|  |  | *C. rugosa* | TAN 0308/57 | JCU | HM018618 |
|  | *Conotrochus* | *C. funicolumna* | SS 022007 stn. 006-056 | JCU | HM018621 |
|  | *Dasmosmilia* | *D.* cf. *lymani* | SS 102005 stn. 25-014 | JCU | HM018625 |
|  | *Dactylotrochus* | *D. cervicornis* | Norfolk 2 stn. DW 2135 | JCU | HM018624 |
|  | *Deltocyathus* | *D. inusitatus* | Norfolk 2 stn. DW 2157 | JCU | HM018626 |
|  |  | *D. magnificus* | SS 102005 stn. 016-012? | JCU | HM018627 |
|  |  | *D. ornatus* | Norfolk 2 stn. DW 2136 | JCU | HM018628 |
|  |  | *D. rotulus* | SS 102005 stn. 25 | JCU | HM018629 |
|  |  | *D. sarsi* | SS 102005 stn. 85-14 | JCU | HM018630 |
|  |  | *D. suluensis* | SS 102005 stn. 171-11 | JCU | HM018631 |
|  | Gen. nov. | sp. nov. | Norfolk 2 stn. DW 2035 | JCU | HM018649 |
|  | *Phyllangia* | *P. papuensis* | SS 102005 stn. 091-044 | JCU | HM018660 |
|  | *Rhizosmilia* | *R. robusta* | Norfolk 2 stn. DW 2135 | JCU | HM018664 |
|  | *Stephanocyathus* | *S. spiniger* | Norfolk 2 stn. CP 2142 | JCU | HM018665 |
|  | *Trochocyathus* | *T. efateensis* | Norfolk 2 stn. DW 2132 | JCU | HM018667 |
|  |  | *T. rhombcolumna* | Norfolk 2 stn. DW 2157 | JCU | HM018668 |
|  | Unidentified | sp. B | Norfolk 2 stn. DW 2135 | JCU | HM018620 |
| Stenocyathidae | *Stenocyathus* | *S. vermiformis* | SS damaged label | JCU | HM018619 |
| Oculinidae | *Madrepora* | *M. oculata* | Norfolk 2 stn. CP 2142 | JCU | HM018659 |
|  | *Cyathelia* | *C. axillaris*** | ? | KU | HM018622 |
| Pocilloporidae | *Madracis* | *M. asanoi*** | ? | KU | HM018656 |
|  |  | *M.* sp. 1**** | ? | KU | HM018657 |
|  |  | *M.* sp. 2**** | ? | KU | HM018658 |
| Fungiidae | *Heliofungia* | *H.* sp. **** | ? | KU | HM018650 |
| Faviidae | *Leptastrea* | *L. transversa*** | ? | KU | HM018655 |
|  | *Platygyra* | *P. sinensis*** | ? | KU | HM018662 |
|  | *Favia* | *F. lizardensis*** | ? | KU | HM018633 |
|  |  | *F. truncatus*** | ? | KU | HM018634 |
| Euphylliidae | *Plerogyra* | *P.* sp. **** | ? | KU | HM018663 |
| Mussidae | *Symphyllia* | *S. valenciennesii*** | ? | KU | HM018666 |
| Sequences from GenBank | | | | | |
| Faviidae | *Barabattoia* | *B. amicorum* | 2 | AB441193 | |
|  | *Caulastraea* | *C. furcata* | 4 | AB117274 | |
|  |  | *C. echinulata* | 7 | FJ345414 | |
|  | *Cladocora* | *C. arbuscula* | 4 | AB117292 | |
|  | *Colpophyllia* | *C. natans* | 4 | AB117228 | |
|  | *Cyphastrea* | *C. serailia* | 4 | AB117257 | |
|  |  | *C. chalcidicum* | 4 | AB117259 | |
|  |  | *C. microphthalma* | 7 | FJ345416 | |
|  | *Diploria* | *D. strigosa* | 4 | AB117225 | |
|  |  | *D. clivosa* | 4 | AB117226 | |
|  |  | *D. labyrinthiformis* | 4 | AB117224 | |
|  | *Diploastrea* | *D. heliopora* | 4 | AB117290 | |
|  | *Echinopora* | *E. pacificus* | 4 | AB117261 | |
|  |  | *E. gemmacea* | 4 | AB117263 | |
|  |  | *E. lamellosa* | 7 | FJ345419 | |
|  | *Favia* | *F. pallida* | 4 | AB117265 | |
|  |  | *F. speciosa* | 2 | AB441194 | |
|  |  | *F. favus* | 4 | AB117267 | |
|  |  | *F. stelligera* | 4 | AB117264 | |
|  |  | *F. leptophylla* | 4 | AB117229 | |
|  |  | *F. fragum* | 8 | AY451351 | |
|  |  | *F. danae* | 7 | FJ345423 | |
|  |  | *F. helianthoides* | 9 | EU371667 | |
|  |  | *F. matthaii* | 9 | EU371671 | |
|  |  | *F. rotumana* | 7 | FJ345428 | |
|  |  | *F. maxima* | 7 | FJ345426 | |
|  | *Favites* | *F. halicora* | 4 | AB117268 | |
|  |  | *F. chinensis* | 4 | AB117269 | |
|  |  | *F. paraflexuosa* | 9 | EU371694 | |
|  |  | *F. abdita* | 9 | EU371687 | |
|  |  | *F. complanata* | 9 | EU371692 | |
|  | *Goniastrea* | *G. aspera* | 4 | AB117271 | |
|  |  | *G. pectinata* | 4 | AB117270 | |
|  |  | *G. deformis* | 2 | AB441195 | |
|  |  | *G. retiformis* | 9 | EU371701 | |
|  |  | *G. australiensis* | 7 | FJ345431 | |
|  |  | *G. edwardsi* | 9 | EU371697 | |
|  |  | *G. favulus* | 9 | EU371698 | |
|  |  | *G. palauensis* | 9 | EU371699 | |
|  | *Leptastrea* | *L. pruinosa* | 2 | AB441196 | |
|  |  | *L. purpurea* | 9 | EU371702 | |
|  | *Leptoria* | *L. irregularis* | 4 | AB117272 | |
|  |  | *L. phrygia* | 4 | AB117273 | |
|  | *Manicina* | *M. areolata* | 4 | AB117227 | |
|  | *Montastraea* | *M. curta* | 4 | AB117278 | |
|  |  | *M. annularis* complex | 4 | AB117260 | |
|  |  | *M. magnistellata* | 4 | AB117279 | |
|  |  | *M. cavernosa* | 8 | AY451356 | |
|  |  | *M. valenciennesi* | 4 | AB117280 | |
|  |  | *M. faveolata* | 8 | AY451357 | |
|  |  | *M. franksi* | 10 | AP008976 | |
|  | *Oulastrea* | *O. crispata* | 2 | AB441197 | |
|  | *Oulophyllia* | *O. crispa* | 4 | AB117275 | |
|  |  | *O. bennettae* | 4 | AB117277 | |
|  | *Platygyra* | *P. daedalea* | 4 | AB117281 | |
|  |  | *P. lamellina* | 4 | AB117282 | |
|  |  | *P. pini* | 9 | EU371722 | |
|  | *Plesiastrea* | *P. versipora* | 3 | AB289561 | |
|  | *Solenastrea* | *S. bournoni* | 4 | AB117291 | |
| Trachyphylliidae | *Trachyphyllia* | *T. geoffroyi* | 4 | AB117287 | |
| Merulinidae | *Scapophyllia* | *S. cylindrica* | 2 | AB441198 | |
|  | *Merulina* | *M. ampliata* | 4 | AB117283 | |
|  |  | *M. scabricula* | 4 | AB117284 | |
|  | *Hydnophora* | *H. exesa* | 4 | AB117285 | |
|  |  | *H. grandis* | 4 | AB117286 | |
| Pectiniidae | *Echinophyllia* | *E. echinoporoides* | 4 | AB117254 | |
|  |  | *E. aspera* | 4 | AB117252 | |
|  |  | *E. orpheensis* | 4 | AB117253 | |
|  | *Pectinia* | *P. alcicornis* | 4 | AB117385 | |
|  |  | *P. paeonia* | 4 | AB117386 | |
|  | *Mycedium* | *M. elephantotus* | 4 | AB117387 | |
|  | *Oxypora* | *O. lacera* | 4 | AB117255 | |
| Mussidae | *Lobophyllia* | *L. corymbosa* | 4 | AB117241 | |
|  |  | *L. hemprichii* | 4 | AB117240 | |
|  |  | *L. pachysepta* | 4 | AB117242 | |
|  | *Symphyllia* | *S. agaricia* | 4 | AB117243 | |
|  |  | *S. radians* | 4 | AB117245 | |
|  |  | *S. recta* | 4 | AB117244 | |
|  | *Scolymia* | *S. vitiensis* | 4 | AB117247 | |
|  |  | *S. cubensis* | 4 | AB117237 | |
|  | *Cynarina* | *C. lacrymalis* | 4 | AB117246 | |
|  | *Acanthastrea* | *A. echinata* | 4 | AB117249 | |
|  |  | *A. rotundata* | 4 | AB117251 | |
|  |  | *A. hillae* | 2 | AB441199 | |
|  | *Mycetophyllia* | *M. danaana* | 4 | AB117323 | |
|  |  | *M. aliciae* | 4 | AB117235 | |
|  | *Isophyllia* | *I. sinuosa* | 4 | AB117238 | |
|  | *Mussa* | *M. angulosa* | 4 | AB117239 | |
|  | *Mussismilia* | *M. braziliensis* | 4 | AB117231 | |
|  |  | *M. harttii* | 4 | AB117232 | |
|  |  | *M. hispida* | 4 | AB117233 | |
|  | *Micromussa* | *M. amakusensis* | 2 | AB441200 | |
|  | *Blastomussa* | *B. wellsi* | 3 | AB289563 | |
| Oculinidae | *Oculina* | *O. diffusa* | 4 | AB117293 | |
|  |  | *O.* sp. | 8 | AY451365 | |
|  | *Galaxea* | *G. fascicularis* | 2 | AB441201 | |
| Euphylliidae | *Physogyra* | *P. lichtensteini* | 3 | AB289562 | |
|  | *Euphyllia* | *E. divisa* | 2 | AB441203 | |
|  |  | *E. glabrescens* | 2 | AB441206 | |
|  |  | *E. ancora* | 2 | AB441204 | |
| Meandrinidae | *Meandrina* | *M. meandrites* | 4 | AB117295 | |
|  |  | *M. brasiliensis* | 4 | AB11797 | |
|  | *Dendrogyra* | *D. cylindrus* | 4 | AB117299 | |
|  | *Dichocoenia* | *D. stokesi* | 4 | AB117298 | |
|  | *Eusmilia* | *E. fastigiata* | 4 | AB117294 | |
|  |  | *E.* sp. | 8 | AY451345 | |
|  | *Ctenella* | *C. chagius* | 2 | AB441208 | |
| Siderastreidae | *Psammocora* | *P. contigua* | 2 | AB441209 | |
|  | *Coscinaraea* | *C. columna* | 2 | AB441210 | |
|  | *Siderastrea* | *S. siderea* | 2 | AB441211 | |
|  |  | *S. radians* | 2 | AB441212 | |
|  |  | *S. stellata* | 2 | AB441213 | |
|  |  | *S. savignyana* | 2 | AB441214 | |
| Agariciidae | *Pavona* | *P. cactus* | 2 | AB441216 | |
|  | *Gardineroseris* | *G. planulata* | 2 | AB441218 | |
|  | *Agaricia* | *A. humilis* | 2 | AB441219 | |
|  |  | *A. agaricites* | 8 | AY451366 | |
|  |  | *A. tenuifolia* | 8 | AY451372 | |
|  |  | *A. fragilis* | 8 | AY451368 | |
|  |  | *A. lamarcki* | 8 | AY451369 | |
|  | *Leptoseris* | *L. cucullata* | 2 | AB441220 | |
|  |  | *L.* sp. | 8 | AY451373 | |
|  | *Pachyseris* | *P. speciosa* | 2 | AB441222 | |
| Fungiidae | *Herpolitha* | *H. limax* | 2 | AB441223 | |
|  | *Fungia* | *F. scutaria* | 2 | AB441224 | |
| Astrocoeniidae | *Stylocoeniella* | *S. guentheri* | 2 | AB441225 | |
|  | *Stephanocoenia* | *S. michelinii* | 2 | AB441228 | |
| Pocilloporidae | *Madracis* | *M. auretenra* | 2 | AB441226 | |
|  | *Pocillopora* | *P. verrucosa* | 2 | AB441230 | |
|  | *Stylophora* | *S. pistillata* | 2 | AB441231 | |
|  | *Seriatopora* | *S.* sp. | 2 | AB441232 | |
|  |  | *S. hystrix* | 2 | AB441234 | |
| Dendrophylliidae | *Tubastraea* | *T. coccinea* | 2 | AB441235 | |
|  |  | *T. aurea* | 2 | AB441237 | |
|  | *Balanophyllia* | *B. elegans* | 15 | DQ445805 | |
|  | *Dendrophyllia* | *D.* sp. | 2 | AB441239 | |
|  | *Turbinaria* | *T. peltata* | 2 | AB441240 | |
| Poritidae | *Goniopora* | *G.* sp. | 2 | AB441241 | |
|  | *Porites* | *P. astreoides* | 2 | AB441242 | |
|  |  | *P. lutea* | 2 | AB441243 | |
|  |  | *P. branneri* | 8 | AY451380 | |
|  |  | *P. divaricata* | 8 | AY451381 | |
|  |  | *P. furcata* | 8 | AY451382 | |
|  |  | *P. porites* | 11 | DQ643837 | |
|  | *Alveopora* | *A.* sp. | 2 | AB441245 | |
| Rhizangiidae | *Astrangia* | *A.* sp. | 11 | NC008161 | |
| Fungiacyathidae | *Fungiacyathus* | *F.* sp. | 2 | AB441255 | |
| Acroporidae | *Acropora* | *A. tenuis* | 5 | AF338425 | |
|  |  | *A. palmata* | 2 | AB441246 | |
|  |  | *A. cervicornis* | 8 | AY451340 | |
|  | *Isopora* | *I. brueggemanni* | 2 | AB441247 | |
|  |  | *I. palifera* | 2 | AB441248 | |
|  |  | *I. togianensis* | 2 | AB441249 | |
|  | *Anacropora* | *A. matthai* | 2 | AB441250 | |
|  |  | *A. forbesi* | 2 | AB441251 | |
|  | *Montipora* | *M. cactus* | 2 | AB441252 | |
|  | *Astreopora* | *A. myriophthalma* | 2 | AB441253 | |
|  |  | *A. explanata* | 2 | AB441254 | |
| Outgroups | | | | | |
| Order | *Genus* | *species* |  |  | |
| Corallimorpharia | *Corynactis* | *C. californica* | 2 | AB441256 | |
|  | *Pseudocorynactis* | *P*. sp. | 2 | AB441258 | |
|  | *Ricordia* | *R. florida* | 2 | AB441260 | |
|  |  | *R. yuma* | 2 | AB441261 | |
|  | *Rhodactis* | *R. mussoides* | 2 | AB441263 | |
|  |  | *R. indosinensis* | 2 | AB441264 | |
|  |  | *R.* sp. | 2 | AB441265 | |
|  | *Actinodiscus* | *A. nummiformis* | 2 | AB441266 | |
|  | *Amplexidiscus* | *A. fenestrafer* | 2 | AB441267 | |
|  | *Discosoma* | *D. carlgreni* | 2 | AB441268 | |
|  |  | *D.* sp. | 2 | AB441269 | |
| Antipatharia | *Cirripathes* | *C.* sp. | 2 | AB441271 | |
| Actiniaria | *Anemonia* | *A.* sp. | 2 | AB441274 | |
|  | *Stichodactyla* | *S.* sp. | 2 | AB441275 | |
| Zoanthidea | *Zoanthus* | *Z.* sp. | 2 | AB441276 | |
|  |  | *Z. kuroshio* | 12 | AB252668 | |
|  | *Sphenopus* | *S. marsupialis* | 2 | AB441277 | |
| Octocorallia | *Acanella* | *A. eburnea* | 13 | EF672731 | |
|  | Keratoisidinae | K. sp. | 14 | EF622534 | |
|  | *Briaerum* | *B. asbestinum* | 11 | DQ640649 | |
|  | *Pseudopterogorgia* | *P. bipinnata* | 11 | DQ640646 | |

*File S1 – Abbreviations*

*Institutions and expeditions*

JCU – James Cook University, Townsville – Australia.

KU – Kyoto University, Kyoto – Japan.

SS and TAN - Collected by the Australia's Commonwealth Scientific and Industrial Research Organisation (CSIRO) in Australian waters.

Bathus and Norfolk - Collected by the Institut de recherche pour le Développement (irD, formerly OrsTOM) and Muséum national d’Histoire naturelle (MNHN) in New Caledonian waters.

*Symbols*

* – Sequences retrieved from GenBank.

** – Sequences provided by Dr. Hironobu Fukami, Kyoto University (KU) – Japan.

*References*

1 – Stolarski et al. (in preparation)

2 – Fukami H, Chen CA, Budd AF, Collins A, et al. (11 authors) (2008) Mitochondrial and nuclear genes suggest that stony corals are monophyletic but most families of stony corals are not (Order Scleractinia, Class Anthozoa, Phylum Cnidaria). PLoS One 3:e3222.

3 – Fukami H, Chen CA, Chiou CY, Knowlton N (2007) Novel group I introns encoding a putative homing endonuclease in the mitochondrial *cox1* gene of scleractinian corals. J Mol Evol 64: 591-600.

4 – Fukami H, Budd AF, Paulay G, Sole-Cava A, Chen CA, et al. (2004) Conventional taxonomy obscures deep divergence between Pacific and Atlantic corals. Nature 427: 832-835.

5 – van Oppen MJH, Catmull J, McDonald BJ, Hislop NR, Hagerman PJ, et al. (2002) The mitochondrial genome of *Acropora tenuis* (Cnidaria; Scleractinia) contains a large group I intron and a candidate control region. J Mol Evol 55: 1-13.

6 – Beagley CT, Okimoto R, Wolstenholme DR (1998) The mitochondrial genome of the sea anemone *Metridium senile* (Cnidaria): Introns, a paucity of tRNA genes, and a near-standard genetic code. Genetics 148: 1091-1108.

7 – Huang D, Meier R, Todd PA, Chou LM (2009) More evidence for pervasive paraphyly in scleractinian corals: systematic study of Southeast Asian Faviidae (Cnidaria; Scleractinia) based on molecular and morphological data. Mol Phylogenet Evol 50(1): 102-116.

8 – Shearer TL, Coffroth MA (2008) Barcoding corals: limited by interspecific divergence, not intraspecific variation. Mol Ecol Resour 8(2): 247-255.

9 – Huang D, Meier R, Todd PA, Chou LM (2008) Slow mitochondrial CO1 sequence evolution at the base of the metazoan tree and its implications for DNA barcoding. J Mol Evol 66(2): 167-174.

10 – Fukami H, Knowlton N (2005) Analysis of complete mitochondrial DNA sequences of three members of the Montastraea annularis coral species complex (Cnidaria, Anthozoa, Scleractinia). Coral Reefs 24(3): 410-417.

11 – Medina M, Collins AG, Takaoka TL, Kuehl JV, Boore JL (2006) Naked corals: skeleton loss in Scleractinia. Proc Natl Acad Sci U S A 103(24): 9096-9100.

12 – Reimer JD, Takishita K, Ono S, Tsukahara J, Maruyama T (2007) Molecular evidence suggesting interspecific hybridization in Zoanthus spp. (Anthozoa: Hexacorallia). Zool Sci 24(4): 346-359.

13 – van der Ham JL, Brugler MR, France SC (2009) Exploring the utility of an indel-rich, mitochondrial intergenic region as a molecular barcode for bamboo corals (Octocorallia: Isididae). Mar Genomics 2: 183-192.

14 – Brugler MR, France SC (2008) The mitochondrial genome of a deep-sea bamboo coral (Cnidaria, Anthozoa, Octocorallia, Isididae): genome structure and putative origins of replication are not conserved among octocorals. J Mol Evol 67(2): 125-136.

15 – Hellberg ME (2006) No variation and low synonymous substitution rates in coral mtDNA despite high nuclear variation. BMC Evol Biol 6(1): 24.
