# Supplementary figures and images for: A Comprehensive Phylogenetic Analysis of the Scleractinia (Cnidaria, Anthozoa) Based on Mitochondrial CO1 Sequence Data
Source: PLoS One. 2010 Jul 8;5(7):e11490. doi: 10.1371/journal.pone.0011490 (PMC2900217; doi:10.1371/journal.pone.0011490)

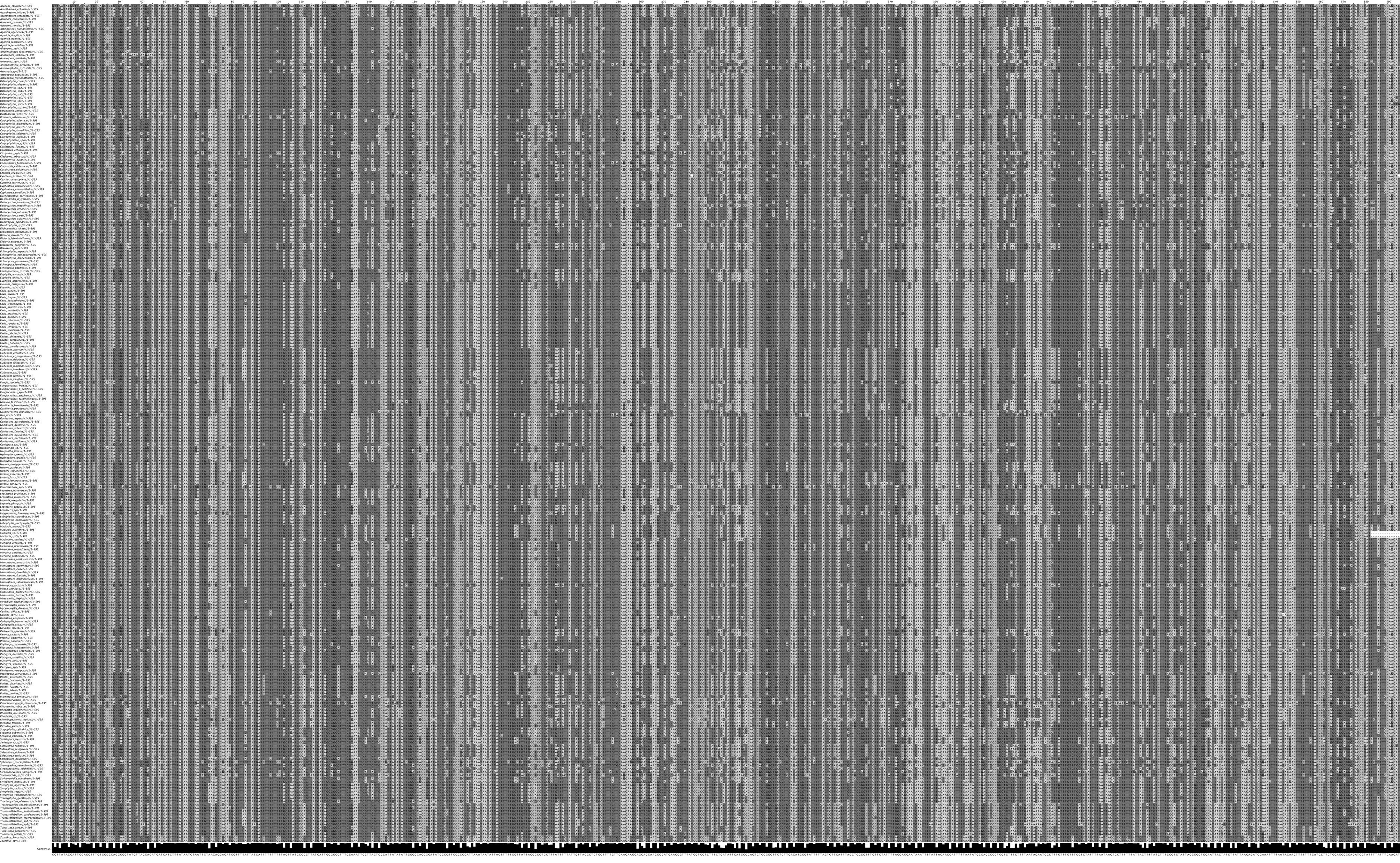

Supplement: File S2 — Partial CO1 gene alignment from 255 anthozoans, including 234 scleractinians from 104 genera representing 25 of the 27 extant families. (9.97 MB TIF) [file pone.0011490.s002.tif]
